# Advanced upper urinary tract urothelial carcinoma is indistinguishable from renal infection by imaging: A case report

**DOI:** 10.1097/MD.0000000000039651

**Published:** 2024-09-13

**Authors:** Jiagui Chai, Runlin Feng, Yuhang Li, Changxing Ke

**Affiliations:** a Department of Urology, The Second Affiliated Hospital of Kunming Medical University, Kunming, China; b Department of Pathology, The Second Affiliated Hospital of Kunming Medical University, Kunming, China

**Keywords:** diagnosis, imaging, symptom, Urinary tract urothelial carcinoma

## Abstract

**Rationale:**

The current diagnostic approach for urinary tract urothelial carcinoma (UTUC) relies on symptoms and imaging. Nevertheless, the diagnosis can be challenging in advanced cases presenting with atypical imaging and symptoms. This article presents an unreported case with atypical imaging and symptoms to provide some experience in diagnosing advanced UTUC.

**Patient concerns:**

A 55-year-old male patient was admitted to the hospital with a 2-month history of persistent left scrotal pain and intermittent left lower back pain.

**Diagnoses:**

Computed tomography and magnetic resonance imaging revealed a left kidney infection. Paradoxically, the patient did not present with a fever, and the white blood cell count was within normal limits. To further clarify the diagnosis, urine cytology was performed. Surprisingly, malignant tumor cells were discovered. The diagnosis of UTUC was considered.

**Interventions:**

The patient underwent radical tumor resection.

**Outcomes:**

The surgery was successfully performed. The patient received regular chemotherapy after surgery. No recurrence was found during the follow-up.

**Lessons:**

This case is a rare and enlightening clinical scenario. When imaging reveals renal infection accompanied by varicocele or renal vein embolism, it is crucial to consider the possibility of advanced UTUC.

## 1. Introduction

Upper urinary tract urothelial carcinoma (UTUC) is a relatively rare malignant tumor that accounts for 5% to 10% of all urothelial carcinomas.^[1]^ The current diagnostic approach for UTUC relies on symptoms and imaging.^[2]^ Hematuria and lower back pain are primary clinical manifestations. Imaging typically reveals a mass with indistinct margins. Nevertheless, the diagnosis can be challenging in advanced cases presenting with atypical imaging and symptoms.^[3]^ This article presents an unreported case with atypical imaging and symptoms to provide some experience in diagnosing advanced UTUC.

## 2. Case presentation

**Figure 1.**
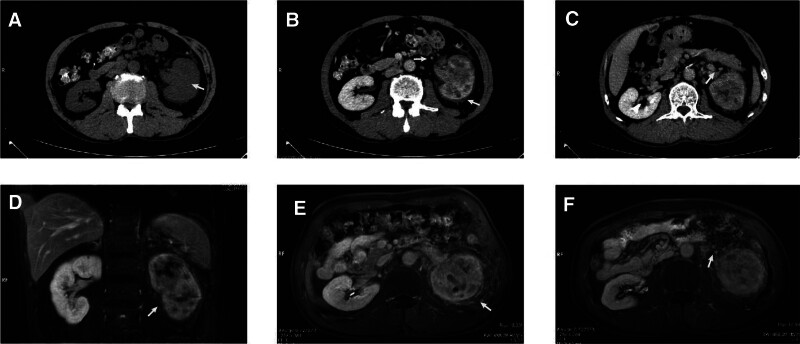
(A) CT showed increased volume of the left kidney, with no mass shadow seen. (B) CT showed thickening of the left perirenal fascia, blurring of the left perirenal fat, and multiple enlarged left retroperitoneal lymph nodes. (C) CT showed a suspected embolism in the left renal vein. (D, E) MRI showed an irregular shape of the left kidney, an uneven signal of the left kidney parenchyma, and a thickening of the perirenal fascia. (F) MRI showed multiple lymph node enlargement in the left retroperitoneum. The white arrows in the figure indicate the location of the lesion. CT = computed tomography, MRI = magnetic resonance imaging.

A 55-year-old male patient was admitted to the hospital with a 2-month history of persistent left scrotal pain and intermittent left lower back pain. Physical examination was unremarkable. Laboratory tests indicated normal urine and blood white blood cell (WBC) counts. Ultrasound identified a left varicocele. Given the patient’s intermittent left lower back pain, computed tomography (CT) was recommended to confirm the presence of other lesions. CT showed an uneven parenchymal density in the left kidney, blurring of the perirenal fat space, thickening of the perirenal fascia, and low-density shadow in the left renal vein (suspected left renal vein embolism) (Fig. 1A–C). Considering that the diagnosis was not fully clear and the resolution of CT might be insufficient, an magnetic resonance imaging (MRI) was recommended. MRI indicated irregular morphology of the left kidney, uneven signals in the parenchyma, thickening of the perirenal fascia, and enlargement of the retroperitoneal lymph nodes (Fig. 1D–F).

Imaging revealed a left kidney infection (no mass was found). Paradoxically, the patient did not present with a fever, and the WBC count was within normal limits. In addition, increased volume of the left kidney, enlarged retroperitoneal lymph nodes, and suspected left renal vein embolism (it might be causatively linked to the left varicocele) were suggested. These could not be fully explained by kidney infection. Therefore, we were caught in a dilemma regarding diagnosis. In this situation, we tried a urine cytology examination to find some diagnostic clues.

Surprisingly, malignant tumor cells (tending to urothelial carcinoma) were discovered by urine cytology. In addition, a cystoscopy was performed (imaging was not available), and no significant abnormalities were found in the bladder and urethra. Therefore, a diagnosis of UTUC was considered. After confirming no distant metastasis, radical surgery was recommended.

## 3. Treatment

**Figure 2.**
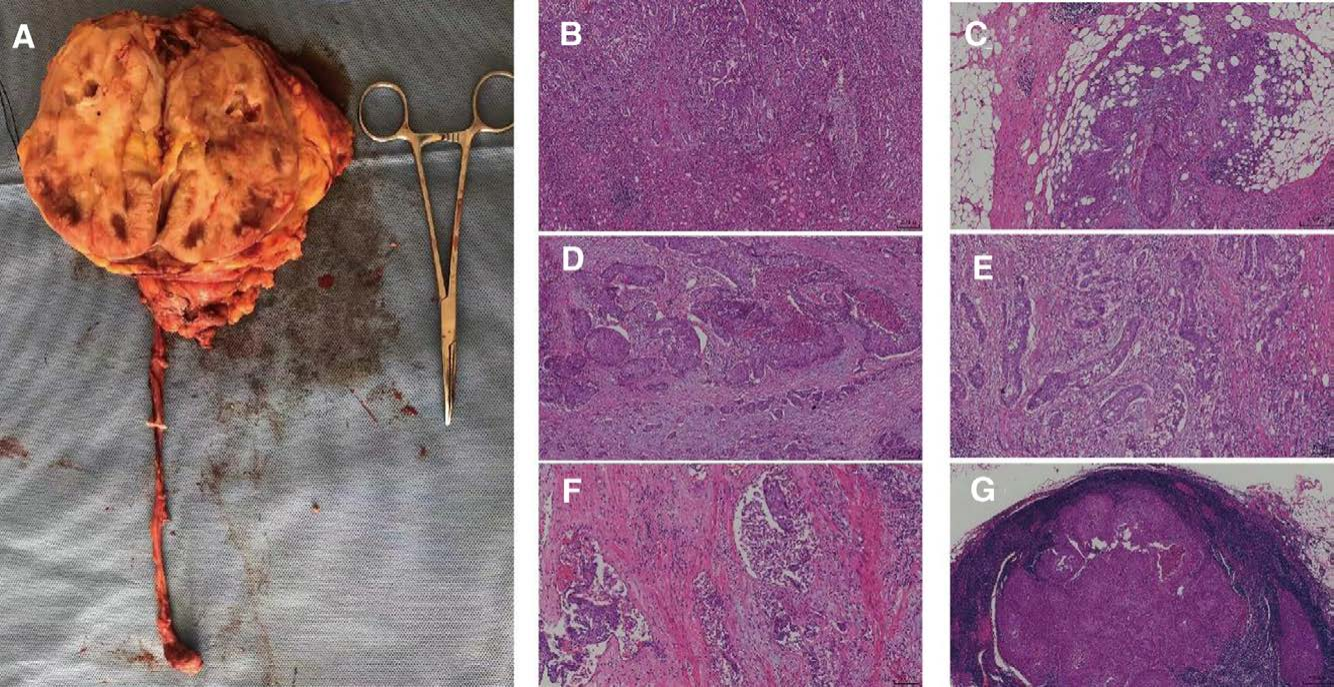
(A) After an incision along the long axis of the kidney, the cut surface appears light yellow with only a small amount of normal renal parenchyma visible. (B) The tumor exhibits invasive growth in renal parenchyma. (C) The tumor invades adipose tissue. (D) The tumor is in a nested distribution. (E) A large number of inflammatory cells can be seen in the tumor interstitium. (F) Cancer thrombus is seen in the vascular system. (G) The tumor with lymph node metastases.

The patient underwent laparoscopic radical nephroureterectomy and lymph node dissection. The scope of surgical resection includes the kidney, ureter, sleeve-shaped bladder, and lymph nodes. Postoperative pathology suggested advanced UTUC (pT_4_N_2_M_x_) (Fig. 2). The patient received 4 cycles of chemotherapy after surgery. To date, the patient has been followed up for 10 months and no recurrence was found.

## 4. Discussion

The primary symptom of UTUC is typically hematuria, which holds diagnostic significance. However, in cases with atypical symptoms, diagnostic challenges may arise.^[2]^ There are reports of UTUC presenting with abdominal pain and fever, which was misdiagnosed as ureteropelvic junction obstruction.^[4]^ In this patient, UTUC presented with scrotal pain, an atypical and rarely reported symptom in UTUC.^[2]^ A reasonable explanation for this symptom may be the formation of a tumor thrombus in the late stage, which blocks the renal vein, thus leading to the obstruction of spermatic vein reflux and causing scrotal pain.^[5]^

Although the imaging of UTUC is evident in most patients,^[2]^ there is still a possibility of confusion with renal infection. It has been reported that a CT scan of xanthogranulomatous pyelonephritis shows ill-defined hyperdense soft tissue in the perinephric space and adjacent to the renal hilum. In addition, MRI reveals ill-defined tissue as iso-to-hypointense to the kidney with scattered cystic or necrotic punctate foci and mild restricted diffusion in the perirenal soft tissue.^[3,6,7]^ The above findings are considered to be confusing between renal infection and UTUC. In the presented patient, the imaging results resembled those of xanthogranulomatous pyelonephritis, adding complexity to the diagnosis of both renal infection and UTUC. We speculate that the difference in the imaging results presented in this case may be related to the extensive infiltration of tumor cells and inflammatory cells.

## 5. Conclusion

In conclusion, this case is a rare and enlightening clinical scenario. When imaging reveals renal infection accompanied by varicocele or renal vein embolism, it is crucial to consider the possibility of advanced UTUC.

## Author contributions

**Writing – review & editing:** Jiagui chai, Runlin Feng.

**Writing – original draft:** Yuhang Li.

**Supervision:** Changxing Ke.
